# Spatial Protein Expression Analysis in Lungs Using Capillary-Based Immunoassay After Laser-Assisted Microdissection

**DOI:** 10.3390/cells15080737

**Published:** 2026-04-21

**Authors:** Stefan Hadzic, Marija Gredic, Vanessa Nebel, Norbert Weissmann, Cheng-Yu Wu

**Affiliations:** Excellence Cluster Cardio-Pulmonary Institute (CPI), Institute for Lung Health (ILH), Universities of Giessen and Marburg Lung Center (UGMLC), German Center for Lung Research (DZL), Justus Liebig University Giessen, Aulweg 130, 35392 Giessen, Germany; stefan.hadzic@innere.med.uni-giessen.de (S.H.); marija.gredic@innere.med.uni-giessen.de (M.G.); vanessa.nebel@uni-giessen.de (V.N.); cheng-yu.wu@innere.med.uni-giessen.de (C.-Y.W.)

**Keywords:** lung injury and repair, protein expression analysis, spatial biology

## Abstract

Unravelling the cellular and molecular mechanisms underlying lung injury and repair requires precise spatial context. Profiling cell-to-cell transcriptional variability and spatial orientation has become increasingly sophisticated, but validating results at the protein level still remains challenging, particularly for low-expressed proteins or small-scale samples. Here, we present a workflow established by our group for spatial protein analysis in the lung by combining two commercially available platforms: (1) laser-assisted microdissection (LMD) with (2) a capillary electrophoretic-based immunoassay (CEI). Using this workflow, we demonstrate a simple, accessible, and sensitive method for spatially capturing regions of interest to investigate small-scale samples or low-expressed proteins. This workflow provides an additional option for orthogonal validation for researchers using omics-based approaches. Furthermore, we validated transcriptome analysis results at the protein level by applying this workflow to a pre-clinical model of cigarette smoke (CS)-induced lung injury. In line with the previous findings, the results showed a significant downregulation of the endothelial cell marker in LMD-enriched alveolar regions, suggesting spatial capillary rarefaction, and activation of the mitogen-activated protein kinase (MAPK) signalling pathway in pulmonary vasculature of CS-exposed mice. Our approach overcomes traditional challenges and provides new opportunities for understanding complex disease pathomechanisms and identifying potential therapeutic targets.

## 1. Introduction

The lung is continuously exposed to a myriad of environmental insults, ranging from airborne pathogens to noxious agents from cigarette smoke (CS) and air pollutants [1,2]. To maintain homeostasis, the lung relies on a sophisticated capacity to repair and regenerate the complex architecture, which comprises branched airways, organised vascular networks, and millions of alveoli [3,4,5,6]. This regenerative potential is governed by precise spatial cues and local signalling niches, such as fibroblast growth factor 10 (FGF10) signalling, which direct cell fate and tissue reconstruction [7,8,9]. However, when the endogenous repair mechanisms are impaired or overwhelmed, diseases such as chronic obstructive pulmonary disease (COPD) and pulmonary fibrosis can develop [7,9].

Pathological remodelling often occurs in discrete regions and involves specific cell populations [9]. Given the structural complexity and the reliance on localised signalling, studying the specific cell types in the lung has proven challenging [10]. Common bulk analyses of tissue homogenates reflect an average expression, which can mask the relevant biological information [11]. Hence, many strategies of sample enrichment have been implemented in recent years to study the distinct phenotypic/genotypic states of the diseases. For instance, laser-assisted microdissection (LMD), a cutting-edge technique, can capture different cell types/compartments from specimen sections [11,12]. This method empowers scientists to generate a hypothesis and elucidate the disease mechanisms [13,14]. In addition, LMD preserves positional information, which can provide unprecedented insight into the spatial biology of individual cells in the organ, and can be used as a method for spatially resolved transcriptomics [9,15].

However, LMD-based experiments have predominantly focused on transcriptome analysis, which does not necessarily correlate with the protein expression level [16]. Moreover, the development of certain diseases relies on protein modifications (e.g., nitration/degradation/glycosylation/phosphorylation), which cannot be pinpointed by transcriptome profiling [13,17]. Traditional Western blotting (WB) has been utilised to investigate LMD-enriched samples, but the WB technique per se is generally labour-intensive and time-consuming [18]. More importantly, it often requires a large amount of sample/protein to achieve an adequate signal-to-noise ratio [19]. Researchers, in particular those studying low-expressing proteins or proteins isolated from small-scale biological samples, may face difficulties when performing conventional WB [20]. Although mass spectrometry is hitherto a powerful tool to investigate proteomics, it is expensive and requires skilled operators [21]. Therefore, there is a need for a simple, accessible, and sensitive technique to validate transcriptome analysis results at the protein level. In this context, our group employed a commercially available automated capillary electrophoretic-based immunoassay (CEI) to analyse protein expression in primary alveolar type II (ATII) epithelial cells isolated from human lungs [22]. This application demonstrated the feasibility of the method for low-input pulmonary samples and provided the rationale for combining LMD with CEI for spatial protein analysis in the lung.

**Figure 1:**
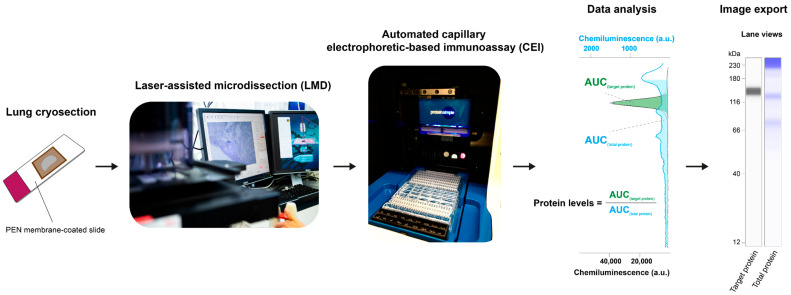
Workflow of spatial protein expression analysis. The cryosections were placed on polyethylene naphthalate (PEN) membrane-coated slides and subjected to laser-assisted microdissection (LMD). Regions of interest in lung sections were collected and loaded on a plate, pre-filled with matrix and reagents. Target protein expression levels were assessed by automated capillary electrophoretic-based immunoassay (CEI). The results were calculated as the ratio of the area under the curve (AUC) between the target protein and the total protein of the entire capillary. The lane view images were exported as a representative picture. Abbreviations: a.u., arbitrary unit.

Against this background, we established a protocol to investigate proteins in LMD-enriched lung tissue compartments using an automated capillary electrophoretic-based immunoassay (Figure 1). This workflow has also been reported for the analysis of phosphoenolpyruvate carboxykinase 2 (PCK2) expression in islets of Langerhans from healthy and diabetic rat pancreas [23]. To the best of our knowledge, our group was the first to apply this workflow to study protein expression in healthy and diseased pulmonary vessels [24]. Here, we extend this approach to additional lung compartments and provide a detailed methodological description of the LMD-CEI workflow for spatial protein analysis in structurally complex lung compartments. The obtained results were validated using immunofluorescence staining on formalin-fixed paraffin-embedded lung tissue sections. Finally, by applying this protocol to a pre-clinical COPD model of CS exposure, we demonstrate that this method enables the precise quantification of cell-type loss and activation of mitogen-activated protein kinase (MAPK) p38 within a spatial context.

## 2. Materials and Methods

### 2.1. Animals

Wild-type mice (C57BL/6N) were obtained from the Charles River Laboratories, Cologne, Germany. Animals were maintained in individually ventilated cages under controlled environmental conditions (22 ± 2 °C; 55 ± 10% humidity; 13 h light cycle) with free access to food and water (ad libitum), bedding, and nesting material. The maximum capacity for housing was five mice per cage. All experimental procedures received ethical approval by the local authorities (Regierungspräsidium Gießen, No. G46/2016, issued on 28 July 2016) and were conducted in full compliance with the Tierschutzgesetz (German Animal Welfare Act) and European Union (EU) Directive 2010/63/EU. Lung tissue from a total of 16 animals was used in this study.

### 2.2. Chronic Cigarette Smoke Exposure

Mainstream cigarette smoke (CS) was generated from 3R4F reference cigarettes (Kentucky Tobacco Research and Development Center, Lexington, KY, USA) with a semi-automatic smoking machine and delivered to the exposure chambers, as reported previously [9]. In this study, a total of 8 animals were exposed to CS for 6 h per day and 5 days per week over 8 months. Particulate matter concentration within the exposure chambers was determined gravimetrically and maintained at 200 mg/m^3^ throughout the study. A corresponding group of age-matched control mice (*n* = 8) was housed under identical conditions but without CS exposure and was designated as the room air (RA) group.

### 2.3. Surgical Instruments

The pre-clinical surgical tools, including tweezers, forceps, Iris scissors, and spring scissors, were purchased from the Fine Science Tools GmbH, Heidelberg, Germany. Prior to harvesting the organs, all the surgical tools were disinfected.

### 2.4. Sampling for Cryopreserved Lungs

Cryopreserved, Tissue-Tek^®^-embedded mouse lungs (*n* = 4 RA; *n* = 4 CS) were cut in 12 µm-thick sections using a cryotome and placed on a glass slide that was covered with a polyethene naphthalate (PEN) membrane (e.g., 11505158; Leica Microsystems GmbH, Wetzlar, Germany). Sections were stained with a 40% haematoxylin solution (diluted with distilled H_2_O, *v*/*v*) for 1 min and dehydrated in the graded ethanol series, as described in Table 1.

### 2.5. Sampling for Paraffin-Embedded Lungs

The paraffin-embedded mouse lungs (*n* = 4 RA; *n* = 4 CS) were cut in 3 µm-thick sections and placed in a warm water bath to remove the fine wrinkles or folds. The sections were then transferred to glass slides (e.g., 25 × 75 × 1 mm, SuperFrost UltraPlus^®^, Epredia, Basel, Switzerland) and stored in a microscope slide box after the sections were dried.

### 2.6. Immunofluorescence Staining and Imaging Using Confocal Microscopy

Slides from paraffin-embedded mouse lungs were deparaffinised and rehydrated following routine protocols, describe in Table 2 and the previous study [13,24].

The slides were then incubated with goat anti-platelet and endothelial cell adhesion molecule 1 (PECAM1; 1:200, AF3628, R&D system, Bio-Techne, Minneapolis, MN, USA), mouse anti-alpha-smooth muscle actin (α-SMA; 1:200, MAB1420, R&D system, Bio-Techne, Minneapolis, MN, USA), and rabbit anti-Pro-surfactant protein C (Pro-SFTPC; 1:500, ab270521, Abcam, Cambridge, UK) antibodies overnight at 4 °C. The next day slides were washed in phosphate-buffered saline and incubated with donkey anti-rabbit Alexa Fluor^®^ Plus 488 (1:400, A32790, Thermo Fisher Scientific Inc., Waltham, MA, USA), donkey anti-goat Alexa Fluor^®^ Plus 555 (1:400, A32816, Thermo Fisher Scientific Inc., Waltham, MA, USA), and donkey anti-mouse Alexa Fluor^®^ Plus 647 (1:400, A32787, Thermo Fisher Scientific Inc., Waltham, MA, USA) for 3 h at room temperature. After washing and staining with 10 ng/mL of Hoechst 33342 (62249, Invitrogen, Thermo Fisher Scientific Inc., Waltham, MA, USA), slides were covered using high-precision microscope cover glasses No. 1.5H (01707242, Paul Marienfeld GmbH & Co. KG, Lauda-Königshofen, Germany) and ProLong™ Glass Antifade Mountant (P36984, Thermo Fisher Scientific Inc., Waltham, MA, USA). The slides were sealed with nail polish CoverGrip™ Coverslip Sealant (23005, Biotium, Fremont, CA, USA) and stored in the dark at 4 °C until imaging. A laser scanning confocal microscope (Leica TCS SP5, Leica Microsystems GmbH, Wetzlar, Germany) was used to examine mouse lung tissue sections, as previously described [9].

### 2.7. Laser-Assisted Microdissection (LMD)

Laser microdissection was performed as described previously with subtle modifications [9]. Briefly, microdissection was performed using an LMD 6000 system (Leica Microsystems GmbH, Wetzlar, Germany) equipped with a laser. Then, 60 frames of bronchi, 80 frames of pulmonary vessels, and 60 frames containing only alveolar septa were dissected from each mouse lung and collected separately in tubes containing 0.1% (*v*/*v*) 10× sample buffer (042-195, ProteinSimple, Bio-Techne, Minneapolis, MN, USA) with a cOmplete™ Mini EDTA-free Protease Inhibitor Cocktail (11836170001, Roche, Merck KGaA, Darmstadt, Germany) in distilled water (total volume was 50 µL). For this amount of LMD-derived tissue, the resulting lysates showed protein concentrations ranging from 0.08 to 0.15 µg/µL, as measured by the Bio-Rad DC Protein Assay (5000111, Bio-Rad, Hercules, CA, USA). Total slide sections were collected as an internal control group. All samples were sonicated 3 times for 30 s using an S30H ultrasonic bath (Elma Schmidbauer GmbH, Singen, Germany) operated at a frequency of 40 kHz and power of 300 Watts, with samples kept in an ice-cold water bath and stored at −80 °C until further processing.

### 2.8. Capillary Electrophoretic-Based Immunoassay (CEI)

Protein samples were prepared following the protocol provided by the manufacturer and as described previously [22]. Briefly, loading samples were prepared in a total volume of 5 µL by mixing LMD-enriched tissue lysates with 1× fluorescence Master Mix containing 40 mM of dithiothreitol (DTT; PS-ST01EZ-8; EZ standard Pack 1; Bio-Techne, Minneapolis, MN, USA). The loading samples were incubated at 95 °C for 5 min and subsequently centrifuged at 16,000× *g* for 2 min. Samples, blocking buffer (043-524, ProteinSimple, Bio-Techne, Minneapolis, MN, USA), primary and secondary antibodies (see below), wash buffer (042-202, ProteinSimple, Bio-Techne, Minneapolis, MN, USA), recasting Replex module (RP-001, ProteinSimple, Bio-Techne, Minneapolis, MN, USA), and Luminol-peroxide mix (PS-CS01, ProteinSimple, Bio-Techne, Minneapolis, MN, USA) were loaded into a plate, pre-filled with separation matrix, stacking matrix, split running buffer 2, and matrix removal buffer (SM-W004, ProteinSimple, Bio-Techne, Minneapolis, MN, USA). Goat anti-PECAM1 (1:10, AF3628, R&D system, Bio-Techne, Minneapolis, MN, USA), mouse anti-α-SMA (1:5, MAB1420, R&D system, Bio-Techne, Minneapolis, MN, USA), rabbit anti-Pro-SFTPC (1:10, ab270521, Abcam, Cambridge, UK), rabbit anti-phospho-p38 MAPK (Thr180/Tyr182; 1:2, #4511, Cell Signaling Technology, Danvers, MA, USA), and anti-p38 MAPK (1:5, #9212, Cell Signaling Technology, Danvers, MA, USA) antibodies were used as primary antibodies. Ready-to-use anti-mouse (042-205, ProteinSimple, Bio-Techne, Minneapolis, MN, USA), anti-rabbit (042-206, ProteinSimple, Bio-Techne, Minneapolis, MN, USA), and anti-goat (043-522-2, ProteinSimple, Bio-Techne, Minneapolis, MN, USA) secondary antibodies were obtained from ProteinSimple. Protein expression was determined by a capillary-based instrument (004-650, JESS, ProteinSimple, Bio-Techne, Minneapolis, MN, USA) following the manufacturer’s instructions. Total protein assay (DM-TP01, ProteinSimple, Bio-Techne, Minneapolis, MN, USA) was performed for protein normalisation purposes. Given that the system automatically loads 50 nL per capillary, an actual protein input ranges 4.0–7.5 ng per capillary. The entire workflow typically requires approximately 6 h from run initiation to data acquisition. The data acquisition, calculation of the area under the curve (AUC), and visualisation of each lane were performed using the Compass for SimpleWestern Version 7.1 (Bio-Techne, Minneapolis, MN, USA). Results were calculated by normalising the AUC of the target protein to total protein in the capillary.

### 2.9. Statistical Analysis

The Prism 10.2.0 software (GraphPad, La Jolla, CA, USA) was used for data analyses. The results were visualised by scatterplots, with the mean values for each group represented by horizontal lines ± standard error of the mean (S.E.M.). All data were logarithmically transformed and assessed for normality using the Prism 10 software, prior to statistical analysis. Visual inspection of quantile–quantile (Q-Q) plots indicated that the data were normally distributed. The two-tailed unpaired *t*-test was used between two groups. For multiple comparisons, Tukey’s honestly significant difference test was used to control the family-wise error rate. An asterisk (*) denotes *p* < 0.05, which was considered statistically significant.

## 3. Results

### 3.1. Mouse Lung Sampling for Spatial Protein Expression Analysis

The maintenance of physiological pressure during sample preparation, namely, vascular perfusion and inflation, is critical for preserving architecture for downstream compartment identification and microdissection [25]. Excessive pressure can cause alveolar destruction or capillary rupture, whereas insufficient pressure can lead to collapse of alveoli and pulmonary vessels [25]. Both scenarios compromise the structural integrity of the tissue. We, therefore, standardised lung preparation as follows.

**Figure 2:**
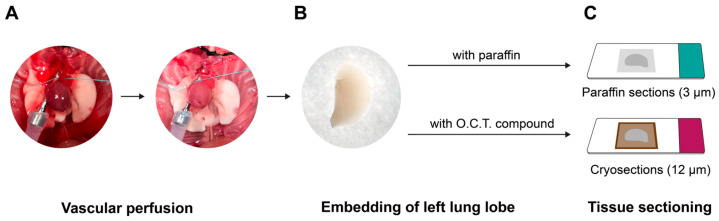
Overview of lung tissue preparation workflow. (**A**) The pulmonary vasculature was flushed with saline solution via arterial perfusion prior to organ harvest. (**B**) The left lung lobe was collected and allocated to one of two downstream embedding procedures. (**C**) Top panel: tissue was fixed in formaldehyde, processed through a graded dehydration series, and paraffin-embedded for sectioning. Bottom panel: the lobe was filled with cryoprotective O.C.T. compound and placed in liquid nitrogen for cryosectioning. Abbreviation: O.C.T., optimal cutting temperature compound.

Following euthanasia, mouse thoracic cavities and abdominal were opened, and the lungs were continuously ventilated with a Minivent device (Type 845; Hugo Sachs Electronic, March, Germany). Vascular perfusion was carried out through the pulmonary artery using 0.9% NaCl solution (*w*/*v*) at 22 cmH_2_O until the lungs were macroscopically blood free (Figure 2A), as described previously [26]. Following removal of the right lung lobes, the left lung lobe (Figure 2B) was processed by one of two methods:

(1) Formalin fixation: The lobe was fixed under concurrent lung inflation (12 cmH_2_O) and vascular perfusion (20 cmH_2_O) with solution containing 3.5–3.7% (*w*/*v*) formaldehyde stabilised with methanol (Otto Fischar GmbH, Saarbrücken, Germany) for 20 min. Following removal from the thoracic cavity, the lung tissue was transferred to a beaker containing formaldehyde solution and stored overnight at room temperature before standard dehydration and paraffin embedding, as previously described [13,24] (Figure 2C, top panel).

(2) Cryopreservation: Tissue-Tek^®^ optimal cutting temperature (O.C.T.) compound was installed through the trachea to fill the left lung lobe. The lung tissue was then removed and placed in a cassette filled with O.C.T. compound. Following wrapping the cassettes with aluminium foil, the samples were immediately frozen in liquid nitrogen and then stored at −80 °C (Figure 2C, bottom panel).

### 3.2. Validation of LMD-Based Spatial Protein Expression Analyses

**Figure 3:**
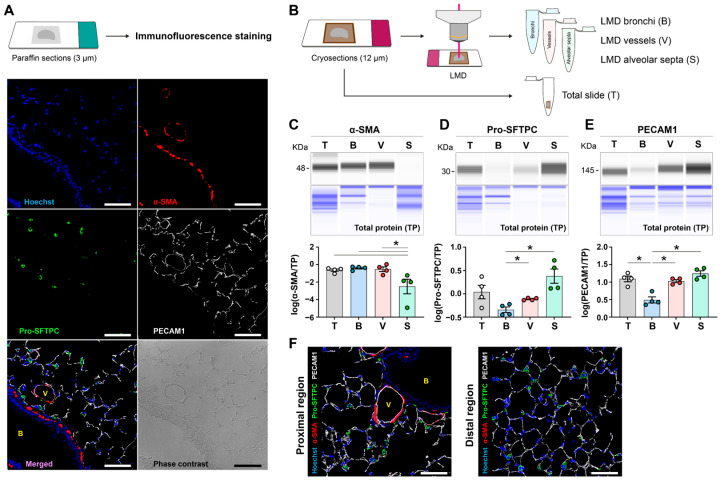
Comparisons between immunofluorescence staining and LMD-based spatial protein expression analyses. (**A**) Localisation of α-SMA (in red), Pro-SFTPC (in green), and PECAM1 (in white), in a formalin-fixed paraffin-embedded mouse lung section. Nuclei were displayed by Hoechst-33342 staining (in blue). The bronchus and vessel are indicated by B and V, respectively. Scale bars = 50 µm. (**B**) Workflow of sampling bronchi (B), pulmonary vessels (V), and alveolar septa (S) using laser-assisted microdissection (LMD) from the cryopreserved, Tissue-Tek^®^-embedded room air (RA)-exposed mouse lung sections (*n* = 4). Entire cryosections were collected as an internal control group, termed as total slide (T). (**C**–**E**) Representative lane view images and protein level quantification results of α-SMA (**C**), Pro-SFTPC (**D**), and PECAM1 (**E**) in entire lung sections and in different lung compartments collected after LMD enrichment, shown in grey. Total protein was used as a loading control, shown in blue. Each lane corresponds to a single capillary, and the band intensity and area under the curve (AUC) are used for quantification. Each dot represents a measurement in a sample obtained from one experimental animal. (**F**) Representative images of proximal and distal lung regions for validations: α-SMA (in red), Pro-SFTPC (in green), PECAM1 (in white), and nuclei staining (in blue). Scale bars = 50 µm. The mean values for each group are represented with a horizontal line ± standard error of the mean (S.E.M.). Data were analysed with appropriate linear models. One-way ANOVA was applied for *p*-value adjustment. Tukey’s honestly significant difference test was used to control the family-wise error rate. *p*-value < 0.05 was considered statistically significant and denoted by an asterisk (*).

We first established the spatial localisation of cell-specific markers using immunofluorescence staining in formalin-fixed paraffin-embedded mouse lungs (Figure 3A). As expected, confocal microscopy revealed distinct spatial patterns. The smooth muscle cell marker, α-SMA (shown in red), was presented in bronchi and pulmonary vessels, with minimal expression in the alveolar tissues. Pro-SFTPC (shown in green) clearly marked the alveolar type 2 epithelial cells in the lungs. PECAM1 (shown in white) was localised to the tunica intima of pulmonary vessels and the capillaries within alveolar septa (Figure 3A). To test whether our protocol could detect proteins on a picogram scale and provide absolute quantitative results that mirrored the immunofluorescence patterns, we further compared the protein levels in LMD-enriched bronchi (B), pulmonary vessels (V), and alveolar septa (S) from mouse lungs with entire cryosections (T; Figure 3B). Our results showed that α-SMA protein levels were significantly enriched in bronchi and vessels (Figure 3C). Pro-SFTPC levels were highly abundant in alveolar septa (Figure 3D). Furthermore, PECAM1 protein was enriched in both pulmonary vessels and alveolar septa but was not in bronchi (Figure 3E). These data were in line with the validations, as shown by the immunofluorescence signals in proximal and distal lung regions (Figure 3F). For instance, immunoreactivity of α-SMA was observed in proximal regions (containing bronchi and large vessels), whereas distal regions (dominated by alveolar septa) showed a barely detectable α-SMA signal (Figure 3F). These data indicated that LMD enrichment is highly specific and validate that our workflow can serve as a rapid, sensitive, and accurate tool for quantifying spatially distinct protein pools.

### 3.3. Potential Applications for Pre-Clinical and Translational Studies

In a recent study, we demonstrated via transcriptome analysis that endothelial nitric oxide synthase (eNOS) expression was decreased in CS-exposed mice compared to room air (RA) controls, a finding we corroborated by counting endothelial cells using immunofluorescence [9]. To demonstrate the utility of the current methodology in disease modelling, we applied the workflow to a murine model of chronic CS exposure. Here, we sought to determine if our LMD-based analysis method could independently validate these findings at the protein level within the specific cell/tissue of interest.

**Figure 4:**
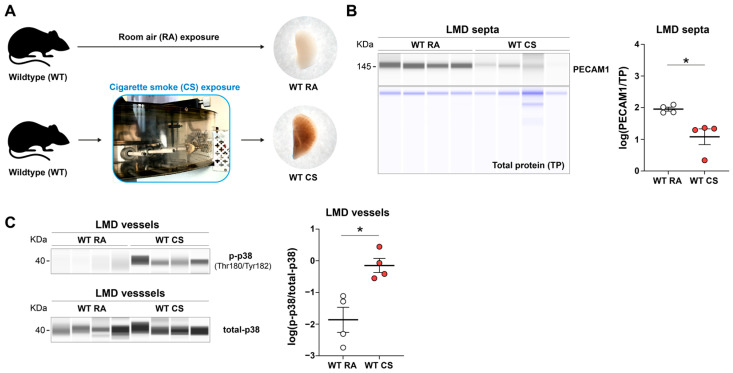
Application of spatial protein expression analyses to a pre-clinical model of lung injury. (**A**) Images of wild-type (WT) mouse lungs after 8 months of room air (RA) or cigarette smoke (CS) exposure. (**B**) Representative lane view images and protein level quantification results of PECAM1 in LMD-enriched alveolar septa (four independent biological replicates per group), shown in grey. Total protein was used as a loading control, shown in blue. (**C**) Representative lane view images and quantification of the protein phosphorylation level of p38 (p-p38) at threonine 180 (Thr180) and tyrosine 182 (Tyr182) in LMD-enriched pulmonary vessels. Total form of protein p38 (total-p38) was used as the reference control. Each lane corresponds to a single capillary, and the band intensity and area under the curve (AUC) are used for quantification. Each dot represents a measurement in a sample obtained from one experimental animal. The mean values for each group are represented with a horizontal line ± standard error of the mean (S.E.M.). Data were analysed with appropriate linear models. The two-tailed unpaired *t*-test was applied for *p*-value adjustment. *p*-value < 0.05 was considered statistically significant and denoted by an asterisk (*).

Wild-type mice were exposed to RA or CS for 8 months (Figure 4A). Following the exposure period, we isolated alveolar septa using LMD to assess the spatial protein expression of the endothelial cell marker PECAM1. Our analysis revealed a significant decrease in the PECAM1 protein level in the LMD-enriched alveolar septa of CS-exposed mice compared to the RA controls (Figure 4B). This finding is in line with our previous observation of capillary rarefaction in CS-induced emphysema [9].

Moreover, in another study, we employed this workflow and provided the evidence that phosphorylation levels of ERK were increased in pulmonary vessels in CS-exposed mice [24]. Here, we further tested whether the same workflow can also detect another MAPK family member, p38 [27]. The results demonstrated increased p38 phosphorylation levels in LMD-enriched pulmonary vessels, further supporting activation of MAPK signalling in this model (Figure 4C).

Overall, these examples support the workflow as a tool to validate transcriptomic findings and to interrogate cell/tissue-specific protein abundance and post-translational modification during lung injury and repair.

## 4. Discussion

Respiratory diseases represent a major cause of global mortality and morbidity [28]. Many lung diseases, including COPD, pulmonary hypertension (PH), interstitial lung diseases (ILD), lung infections, and lung cancer, are characterised by regional heterogeneity, in which pathological alterations can be confined to discrete micro-niches [24,29,30,31,32]. Accordingly, spatially resolved protein profiling approaches are particularly relevant for dissecting disease mechanisms and advancing therapeutic strategies [33].

Several spatial proteomics platforms provide high-dimensional readouts of proteome profiles. For instance, the NanoString GeoMx digital spatial profiler (DSP) enables multiplex region-of-interest (ROI)-based profiling of proteins using photocleavable oligonucleotide tags, typically across regions spanning approximately 1 to ~5000 cells [34]. This system has been widely applied to study spatial protein profiles in different lung diseases, such as severe acute respiratory syndrome coronavirus 2 (SARS-CoV-2) pulmonary infection [30], non-small-cell lung cancer (NSCLC) [31], COPD [29], and ILD [32]. Another example is co-detection by indexing (CODEX)/PhenoCycler, which provides highly multiplexed cyclic imaging with DNA-conjugated antibodies and has been demonstrated up to ~60 markers in situ, including applications in cancer immunotherapy response analyses [35,36,37]. Besides, the Imaging Mass Cytometry system has been used in spatial mapping of lung pathology in patients with SARS-CoV-2 or with other lung infections [38]. This system can multiplex single-cell protein imaging at subcellular resolution [39]. In addition, the system of nanodroplet processing in one pot for trace samples (nanoPOTS) is a mass-spectrometry-based workflow that can quantify hundreds to thousands of proteins from LMD-enriched tissues with protocols reporting expected outcomes of >3000 proteins at 50 μm spatial resolution depending on sample type and instrumentation of mass spectrometry [40,41]. However, these platforms require specialised operators, instrumentation, and/or computational infrastructure [21]. Furthermore, although these systems offer extensive proteome coverage, the ability to quantify specific protein targets in a consistent manner may be limited by factors such as detection sensitivity or stochastic sampling in mass-spectrometry-based approaches [21]. Consequently, targeted validation assays or experiments are required to support conclusions [41].

In this context, the current workflow demonstrates the feasibility of LMD-based spatial protein expression analysis and provides a solution to validate protein targets from limited source material. The total protein assay was used as the normalisation method to avoid reliance on a single internal control protein, the measurement of which may be influenced by antibody performance and tissue-specific expression variability [42]. LMD-enriched samples, in this study, contained 0.08–0.15 µg/µL of protein and were prepared in a 5 µL total volume, corresponding to 0.40–0.75 µg of protein per sample. Since the instrument of CEI automatically loads only 50 nL into each capillary [42], the actual protein input per capillary in the present workflow was approximately 4.0 to 7.5 ng (up to 5000-fold less protein input than conventional WB). Based on these yields, up to 50-fold more LMD-enriched samples would be required to reach the protein input commonly used for conventional WB (standard loading is usually 20 µg of protein per lane) [42]. Hence, compared with traditional WB, the capillary electrophoretic-based immunoassay substantially reduces hands-on time, improves technical reproducibility, and requires markedly lower protein input, making it well suited for LMD-derived samples where protein yields are inherently limited [18,19,20].

By combining two commercially available platforms, the LMD-CEI workflow offers several advantages, including low protein input requirements and a relatively low instrumentation barrier for laboratories seeking targeted validation in spatially defined tissue regions. However, it is inherently low plex, depends on the availability and performance of suitable antibodies, and does not provide unbiased discovery-level proteome coverage. In addition, several technical considerations merit attention. Preservation of lung architecture during perfusion and inflation is critical to ensure reproducible sampling of comparable anatomical regions. Antibody selection and validation remain essential, as assay performance can vary between targets and may be influenced by epitope accessibility, fixation-related changes, or cross-reactivity. Lung tissue also poses specific practical challenges for LMD-based workflows. Air-filled alveolar spaces can reduce targeting efficiency and increase the time required for LMD [43]. Likewise, alveolar septa are extremely thin structures and may yield substantially lower amounts of protein than bronchial or vascular compartments, thereby requiring larger capture areas or increased sampling frames. Furthermore, differences in extracellular matrix (ECM) composition across lung compartments may influence protein extraction and lysis efficiency [44]. For instance, structural ECM or matrisome proteins may require different or more stringent extraction conditions for efficient recovery [45]. In this regard, samples in the present study were prepared using a sodium dodecyl sulphate (SDS)-containing sample buffer, and the analysed lysates mainly represented the extractable/soluble protein fraction. Thus, the influence of ECM composition on protein recovery and on the interpretation of normalisation should be considered when comparing different compartments. Despite these limitations, this approach offers a practical means to investigate physiological and pathological mechanisms in organs with structural complexity, including the lungs. This is particularly relevant for lung injury and repair, where spatially restricted alterations in signalling, cell-type composition, or phenotypic switching may occur in discrete anatomical compartments.

Future applications of this workflow may include expansion to simultaneously investigate additional protein targets by multiplexing with infrared (IR) and near-infrared (NIR) channels, as well as application to other structurally complex tissues and disease models. In addition, artificial intelligence (AI)-assisted image analysis and region selection (e.g., using Aivia software) may further improve LMD targeting efficiency, reduce operator-dependent variability, and enhance throughput. The workflow may also be applied in routine laboratory practice. For instance, one can validate alterations in compartment-specific protein patterns identified in immunohistochemistry or immunofluorescence images through quantitative analysis by LMD-CEI in the corresponding microdissected regions. Furthermore, the workflow may support biomarker identification, validation of data derived from spatial RNA sequencing or spatial proteomics analyses, and the mapping of signalling alterations, such as changes in phosphorylation status, that contribute to impaired repair, remodelling, or disease progression [24].

Our proof-of-concept application illustrates the ability of this method to measure cell-type loss and/or phenotypic switching in a spatial context. This capability is particularly important for studying repair failure, where depletion or dysfunction of specific progenitor or vascular populations may be restricted to defined anatomical locations. The innovative aspect of the present study lies in the application and methodological refinement of a combined LMD-CEI workflow for targeted protein analysis in structurally complex lung compartments. Overall, this workflow expands the toolkit for spatial protein analysis and supports a more comprehensive understanding of lung disease pathomechanisms and therapeutic target identification.

## 5. Conclusions

In this article, we established and validated a protocol for spatial protein expression analysis by coupling LMD with automated capillary electrophoretic-based protein immunoassay. This approach addresses a critical gap in current methodologies by enabling the quantification of proteins from spatially defined regions, which is often unfeasible with traditional WB due to sample volume requirements. We demonstrated the high sensitivity and specificity of this technique by accurately profiling cell-type-specific markers in the lung and further validated its application in a pre-clinical model of CS-induced lung injury. This workflow not only serves as a powerful tool for validating spatial transcriptomic/proteomic data but also empowers researchers to investigate disease mechanisms and identify therapeutic targets in situ with high precision.

## Figures and Tables

**Table 1:** Staining and dehydration protocol for laser-assisted microdissection (LMD).

Step	Time (Seconds)	Reagent	Manufacturer
1	10	Absolute ethanol	108543, Merck KGaA, Darmstadt, Germany
2	60	Haematoxylin solution 40%	CATHE-M, Biocare Medical LLC, Concord, CA, USA
3	15 (2×)	Ethanol 70%	ETO-5000-70-1, SAV Liquid Production GmbH, Flintsbach am Inn, Germany
4	15 (2×)	Ethanol 96%	27695, Otto Fischar GmbH, Saarbrücken, Germany
5	30 (2×)	Absolute ethanol	108543, Merck KGaA, Darmstadt, Germany

**Table 2:** Deparaffinisation and rehydration protocol for paraffin-embedded slides.

Step	Procedure	Time (Minutes)	Reagent/Condition	Manufacturer
1	Deparaffinisation	60	59 °C (heating oven)	UFB400, Memmert, Schwabach, Germany
2		10 (3×)	Xylol	Carl Roth GmbH&Co.KG, Karlsruhe, Germany
3	Rehydration	5 (2×)	Ethanol 99.6%	27694, Otto Fischar GmbH, Saarbrücken, Germany
4		5	Ethanol 96%	27695, Otto Fischar GmbH, Saarbrücken, Germany
5		5	Ethanol 70%	ETO-5000-70-1, SAV Liquid Production GmbH, Flintsbach am Inn, Germany
6		3 (2×)	Distilled water	3478.1, Carl Roth GmbH&Co.KG, Karlsruhe, Germany

## Data Availability

The original contributions presented in this study are included in the article. Further inquiries can be directed to the corresponding author.
